# Research trends and hotspots of health-related quality of life: a bibliometric analysis from 2000 to 2019

**DOI:** 10.1186/s12955-021-01767-z

**Published:** 2021-04-23

**Authors:** Si Zheng, Anqi He, Yan Yu, Lingling Jiang, Jing Liang, Peigang Wang

**Affiliations:** 1 School of Health Sciences, Wuhan University, NO. 115 Donghu Road, Wuhan City, 430071 China; 2 Wuhan Library, Chinese Academy of Science, NO. 25 West of XiaoHonghan, Wuhan City, 430071 China

**Keywords:** Health-related quality of life, Bibliometric, Scientific output, Keyword clustering

## Abstract

**Background:**

The number of research articles on health-related quality of life (HRQoL) has been strikingly increasing. This study aimed to explore the general trends and hotspots of HRQoL.

**Methods:**

Based on the Web of Science database, research on HRQoL published between 2000 and 2019 were identified. A bibliometric analysis was performed based on the number of articles, citations, published journals, authors' addresses, and keywords. Descriptive analysis, visualization of geographic distribution and keyword clustering analysis were applied to the collected data.

**Results:**

The annual number of articles showed growth over the past twenty years, but the annual total citations and annual citations per article were both in decreasing trends. Articles about HRQoL were more likely to be published in journals of multi-subject categories. The HRQoL research was mainly distributed across North America and Europe throughout the twenty years and ushered in a vigorous development worldwide after 2015. Cooperation strength between domestic institutions was much greater than that of international institutions. HRQoL research had six concentrated clusters: HRQoL, Depression, Obesity, Disability, Oncology, Fatigue.

**Conclusion:**

This study provided an overall perspective of global research trends and hotspots in HRQoL, and a potential insight for future research. HRQoL research had experienced significant increasing development during 2000–2019, especially the HRQoL measurement instruments, however, there were significant regional disparities in scientific output in HRQoL.

## Background

The Health-Related Quality of Life (HRQoL) is derived from the concept of Quality of Life (QoL), consisting of the physical, psychological and social health dimensions [1]. Initially, the QoL was a sociological concept defined by American economist J. K. Calbraith in the 1950s [2]. After that, the concept of QoL gradually extended to the medical field. Since HRQoL measured the disease and treatment outcome from the patient's point of view, it was rapidly gaining acceptance as a measurable health outcome and became an important component of health surveillance [3]. In the 1980s, the concept of HRQoL had evolved to encompass those aspects of overall quality of life that can be clearly shown to affect health, either physical or mental [4].

Plenty of instruments were developed to measure and evaluate the status of HRQoL scientifically. Some of them were generic instruments, for example, the EuroQol Group developed the EuroQol Five Dimensions Questionnaire (EQ-5D) in 1990 based on a descriptive system that defined health in terms of 5 dimensions: Mobility, Self Care, Usual Activities, Pain/Discomfort, and Anxiety/Depression [5, 6]. The 36-item short-form (SF-36) was constructed in 1992 to survey health status in the Medical Outcomes Study [7], which yielded an eight-scale profile of scores as well as physical and mental health summary measures [8]. These instruments have been widely tested and used in both general population and patient samples [8–10]. Specific instruments have also achieved great development. Such as the Pediatric Quality of Life Inventory (PedsQL) 4.0 Generic Core Scales for children [11], and the Medical Outcomes Study HIV (MOS-HIV) Health Survey for HIV/AIDS [12].

At the beginning of the twenty first century, HRQoL measurement has emerged as an essential health outcome in clinical trials, clinical practice improvement strategies, and health care services research and evaluation [13]. What's more, the international interest on HRQoL has continued to grow. Researchers from Australia, Canada, Europe, Japan, and the United States established the International Quality of Life Assessment (IQOLA) project to translate and validate the HRQoL instruments for international utilization [14]. Based on the continuous development of HRQOoL theory and instruments, researchers and practitioners in fields outside medical and public health fields such as sociology, psychology, and social work were also actively engaged in HRQoL research [3].

Research on HRQoL was carried out in many fields worldwide and generated a large amount of research literature. However, the general trend and impact of the research production on the HRQoL topic was not yet documented. The measurement of research output and their impact were multidimensional and complex [15]. As an important tool, bibliometric analysis can comprehensively measure research literature's influence and production on a specific subject [16], utilizing many indicators including impact factor (IF), the total number of articles, the total number of citations, authorship, and researchers' or institutions' collaboration network among others [17].

Therefore, in this study, the bibliometric analysis of published HRQoL articles for the period from 2000 to 2019 was performed to (a) summarize global research trends in terms of the number of articles published, the distribution of journals, countries and institutions, and the frequency of keywords; (b) figure out the hotspots and potential future directions in HRQoL research; (c) provide recommendations on practice and policy to promote HRQoL research.

## Methods

### Data collection

**Fig. 1:**
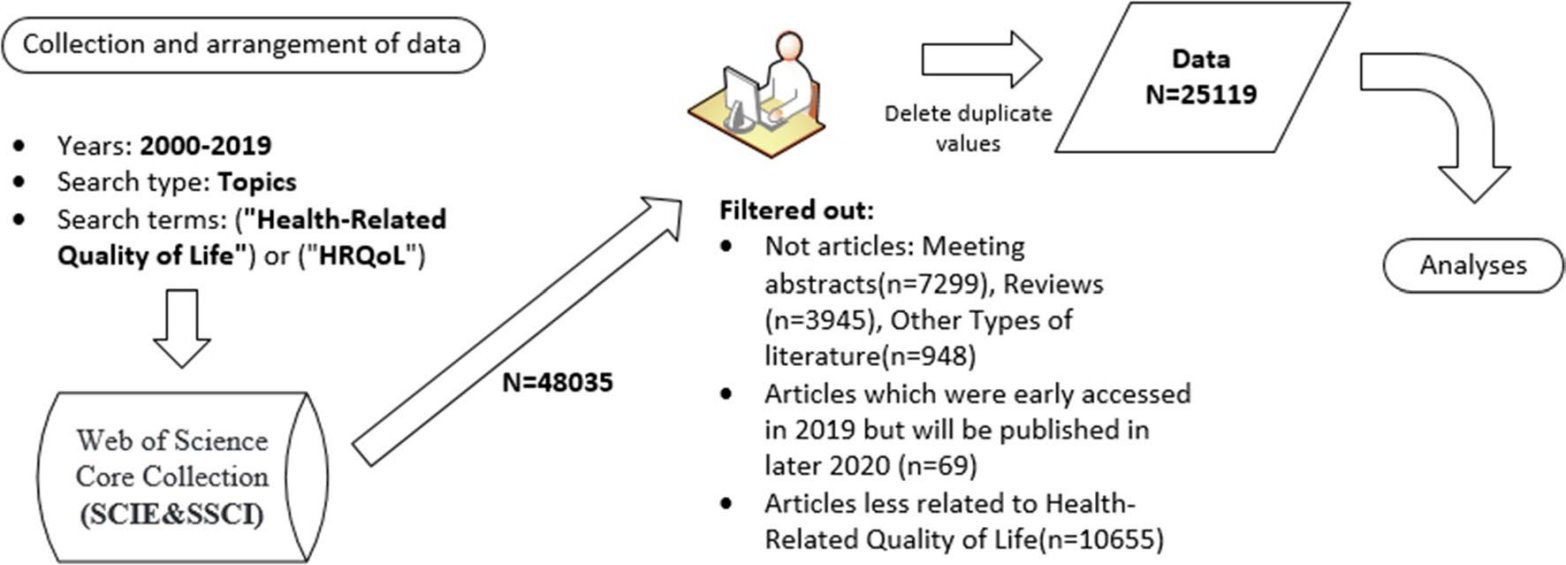
The process of data collection on HRQoL researches

Data used in this study were retrieved from the WOS Core Collection, the online version of the Science Citation Index-Expanded (SCIE) and the Social Sciences Citation Index (SSCI). The search strategy was developed by querying the term ("Health-Related Quality of Life") or ("HRQoL") as topics in the WOS database. All publications which had been published between 2000 (1st January) and 2019 (31st December) were included. The search process was carried out between March 24 and 30, 2020 (Fig. 1). From 2000 to 2019, a total of 48,035 publications related to HRQoL were published. Our analyses were limited to only articles. We eliminated 69 articles which publishing years were marked as 2020 (early accessed in 2019). Then we reviewed the titles and keywords of the rest articles and filtered out 10,655 articles that were found to be less related to our topic. After that, we used the “Delete Duplicate Value” function of Excel to eliminate duplicates in samples. Finally, 25,119 articles were included for the analyses in the scope of bibliometrics.

### Analyses

Four types of analysis were conducted. Firstly, Microsoft Excel 2013 was used to calculate and rank the number of published articles and citations, from which the general trend of HRQoL research was derived. Secondly, the authors' addresses were contained in the *C1* field of the collected database. We did geography and collaboration analysis according to the authors' addresses. We used Python as a geocoding tool to find the longitude and latitude of each address, then used ArcGIS 10.3 to visualize the worldwide geographic distribution of authors. Articles from England, Scotland, Northern Ireland, and Wales were unified as articles from the UK, articles from Taiwan were treated as articles from China.

Thirdly, the cumulative impact of an institution's scientific output was measured by the index *h*, defined as the number of papers with citation number ≥ *h* [18], the one with the higher *h* is likely to be the more accomplished institution.

Finally, the keyword analysis took author keywords as objects author keywords provided a reasonable description of an article's theme and offered information on issues that researchers were concerned about [17]. Before analysis, we merged some synonymous keywords. For instance, "Health-related Quality of life," "health related quality of life," "HRQOL," "health-related quality of life (HRQOL)" and other synonymous keywords were merged into "Health-related Quality of life/HRQoL." The keyword cluster analysis was conducted in two steps. Firstly we used CiteSpace 5.6.R3 to find the keyword clusters in HRQoL research, which can group the most closely related keywords into a cluster based on the connections between the keywords of the articles, and those clusters were ranked by the most frequently used keyword in articles [15]. Secondly, we used our specialized knowledge to optimize the clustering of keywords and come to a conclusion. Keyword clusters can be used to analyze the hotspot in the research field [17] and examine trends in research topics [16].

Then we treated every five years as a period (2000–2004, 2005–2009, 2010–2014, 2015–2019), numbering in chronological order (i = 1,2,3,4). We calculated the sequential growth rate (SGR) for each period to compare the heat of the top 100 most frequently used keywords in the recent period with the period preceding it, and selected quickly rising terms of HRQoL research. The SGR was calculated based on the following formula:

$${\text{SGR}} = \frac{{\left( {N_{i} - N_{i - 1} } \right)}}{{N_{i - 1} }}$$

in which $N_{i}$ is the number of articles published in the period *i*. When SGR greater than 0, the heat of the keyword increased during this period. The greater the SGR, the faster the heat of the keyword have increased during this period. Using surged topical terms rather than the most frequently occurring title words is particularly suitable for detecting emerging trends and abrupt changes [19].

## Results

### General trend

**Fig. 2:**
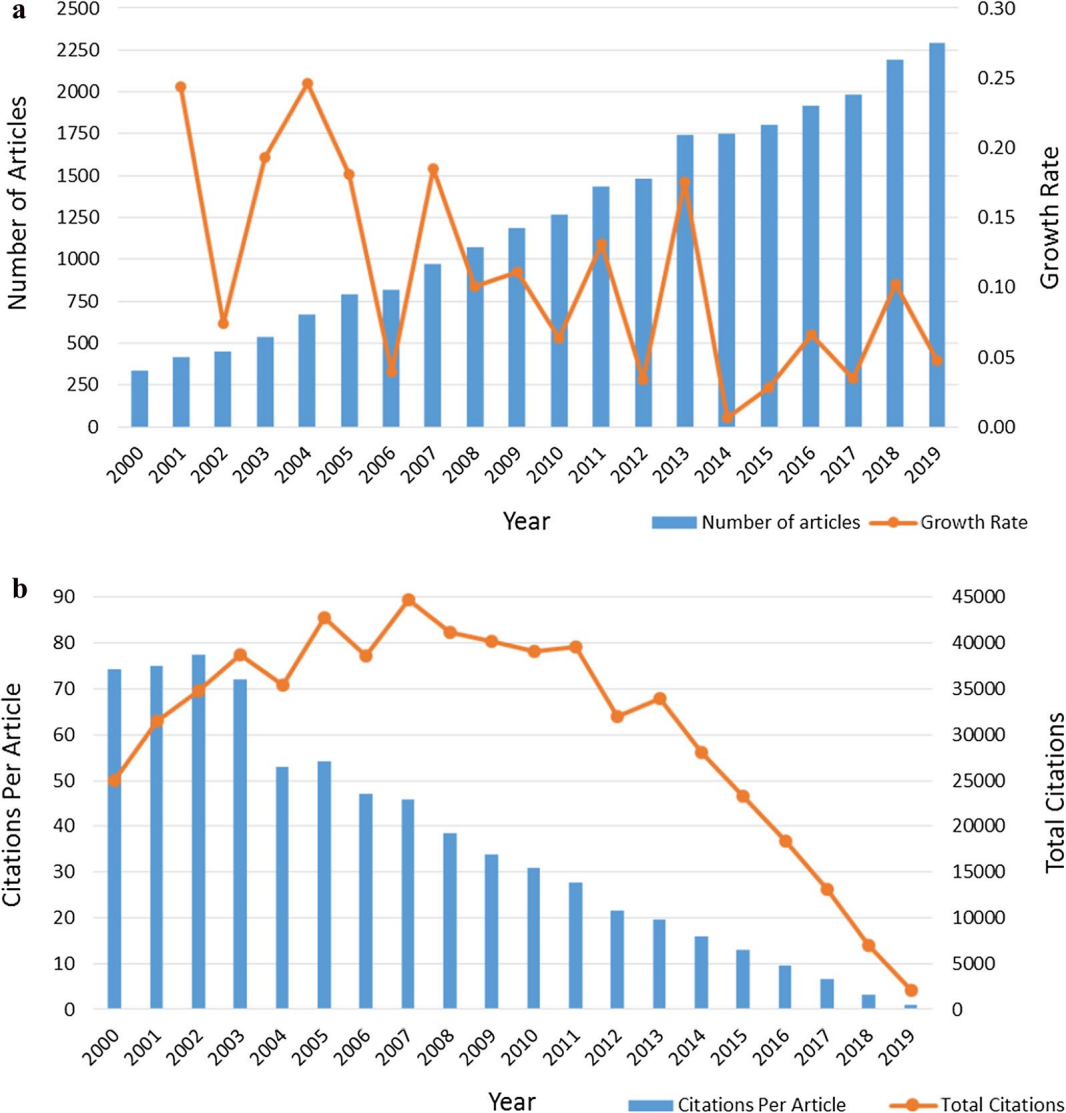
Trends of HRQoL articles and citations from 2000 to 2019

The number of articles about HRQoL and citations over 2000–2019 counted and displayed in Fig. 2. Among them, Fig. 2a presented the trend of the number of articles and the annual growth rate and showed that the number of HRQoL studies had increased continuously over the years, the yearly number of HRQoL articles risen from 337 in 2000 to 2294 in 2019. The growth rate fluctuated significantly in the twenty years, but overall, the last six-year period's growth rate was lower than before. Figure 2b showed the trend of annual total citations (TC) and annual citations per article (CPA), the trend of TC was nearly an inverted U-shaped and had a turning point in the year 2007.

### Journal analysis

From 2000 to 2019, 25,119 articles were published in 2565 journals. The top twenty most productive journals on HRQoL were listed in Table 1, most of the journals fall into two or more categories at once. Articles about HRQoL were more likely to be published in journals of multi-subject categories, showed that HRQoL has developed into a multidisciplinary field. Public, Environmental and Occupational Health and Health Care Sciences and Services were the top two categories, indicating they exerted a wider influence in the HRQoL field. In addition, Cancer had the highest five-year impact factor (6.412) with 131 articles, followed by Value in Health (6.131) with 203 articles. When examining citations, Medical Care had the highest CPA (127.79), consistent with the results in the Table 5 in “Appendix 1”, Medical Care published three articles in the most cited ten articles.

**Table 1:** Top 20 journals with the most published literature on HRQoL

Journal title	TA(R)	CPA	JCR categories	IF(5 year)
Quality of Life Research	1477(1)	27.48	P, E&OH; HCS&S; HP&S	2.979
Health and Quality of Life Outcomes	844(2)	19.57	HCS&S; HP&S	3
PloS One	296(3)	10.51	Multidisciplinary Sciences	3.337
Value in Health	203(4)	30.87	Economics; HCS&S	6.131
Supportive Care in Cancer	185(5)	16.77	Oncology; Rehabilitation; HCS&S	3.002
Psycho-Oncology	153(6)	25.12	PM; Oncology; SS, B; Psychology	4.115
Spine	153(6)	30.12	CN; Orthopedics	3.616
BMC Public Health	143(8)	19.2	P,E&OH	3.275
Cancer	131(9)	50.92	Oncology	6.412
Epilepsy & Behavior	127(10)	19.27	CN; BS; Psychiatry	2.677
Journal of Urology	126(11)	40.56	U&N	4.961
BMJ Open	116(12)	5.5	M,G&I	2.863
Community Dentistry and Oral Epidemiology	113(13)	49.09	P,E&OH; D,OS&M	2.778
Journal of Clinical Epidemiology	113(13)	59.35	P,E&OH; HCS&S	5.645
Disability and Rehabilitation	112(15)	16.72	Rehabilitation	2.311
International Journal of Environmental Research and Public Health	106(16)	5.32	P,E&OH; Environmental Sciences	2.948
Respiratory Medicine	106(16)	31.33	C&CS; Respiratory System	3.702
Journal of Rheumatology	100(18)	41.14	Rheumatology	3.774
Medical Care	100(18)	127.79	P,E&OH; HCS&S; HP&S	3.991
Archives of Physical Medicine and Rehabilitation	99(20)	36.19	Sport Sciences; Rehabilitation	3.618
Journal of Psychosomatic Research	99(20)	34.53	Psychiatry	3.311

*TA(R)* total articles of each journal (rank in all articles), *CPA* citations per article, *JCR categories* categories in journal citation reports, *IF(5 year)* 5-year impact factor, *P, E&OH* public, environmental & occupational health, *HCS&S* health care sciences & services, *HP&S* health policy & services, *PM* psychology, multidisciplinary, *SS, B* social sciences, biomedical, *CN* clinical neurology, *BS* behavioral sciences, *U&N* urology & nephrology, *M, G&I* medicine, general & internal, *D, OS&M* dentistry, oral surgery & medicine, *C&CS* cardiac & cardiovascular systems

### Geography and collaboration analysis

**Fig. 3:**
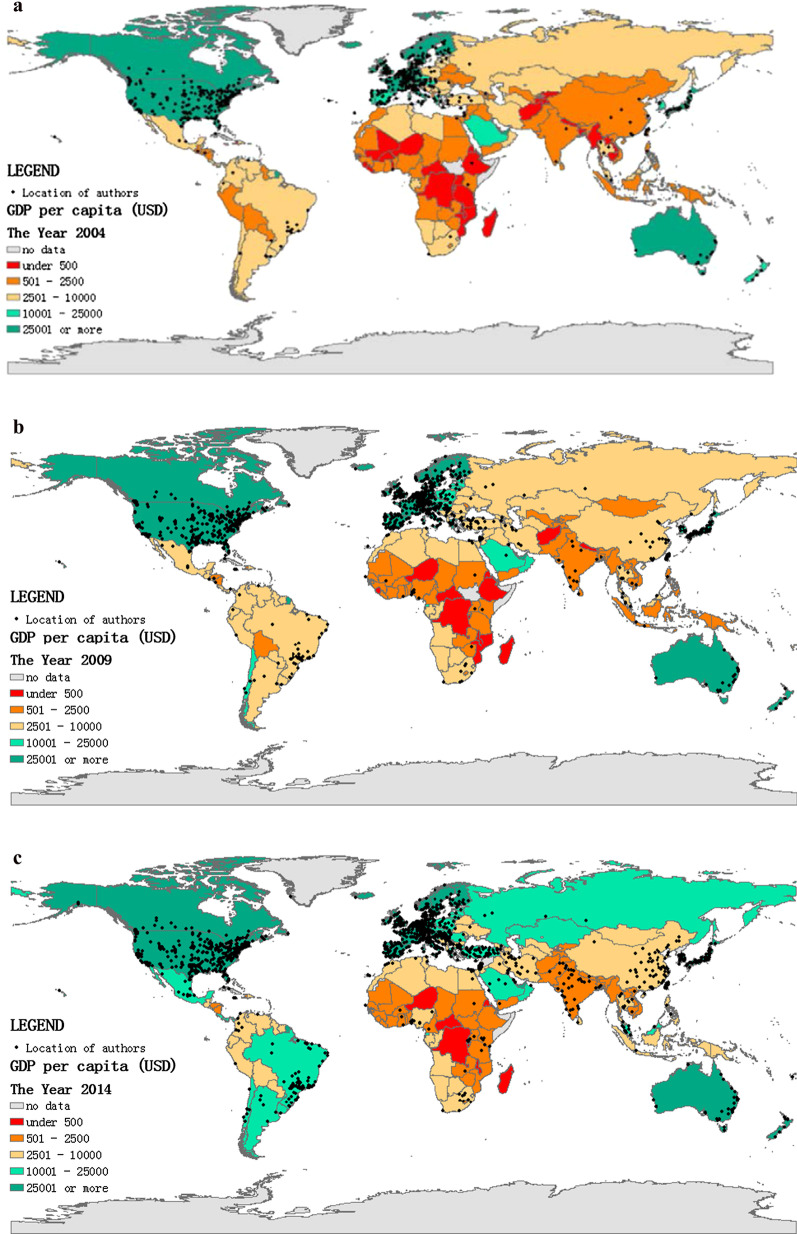

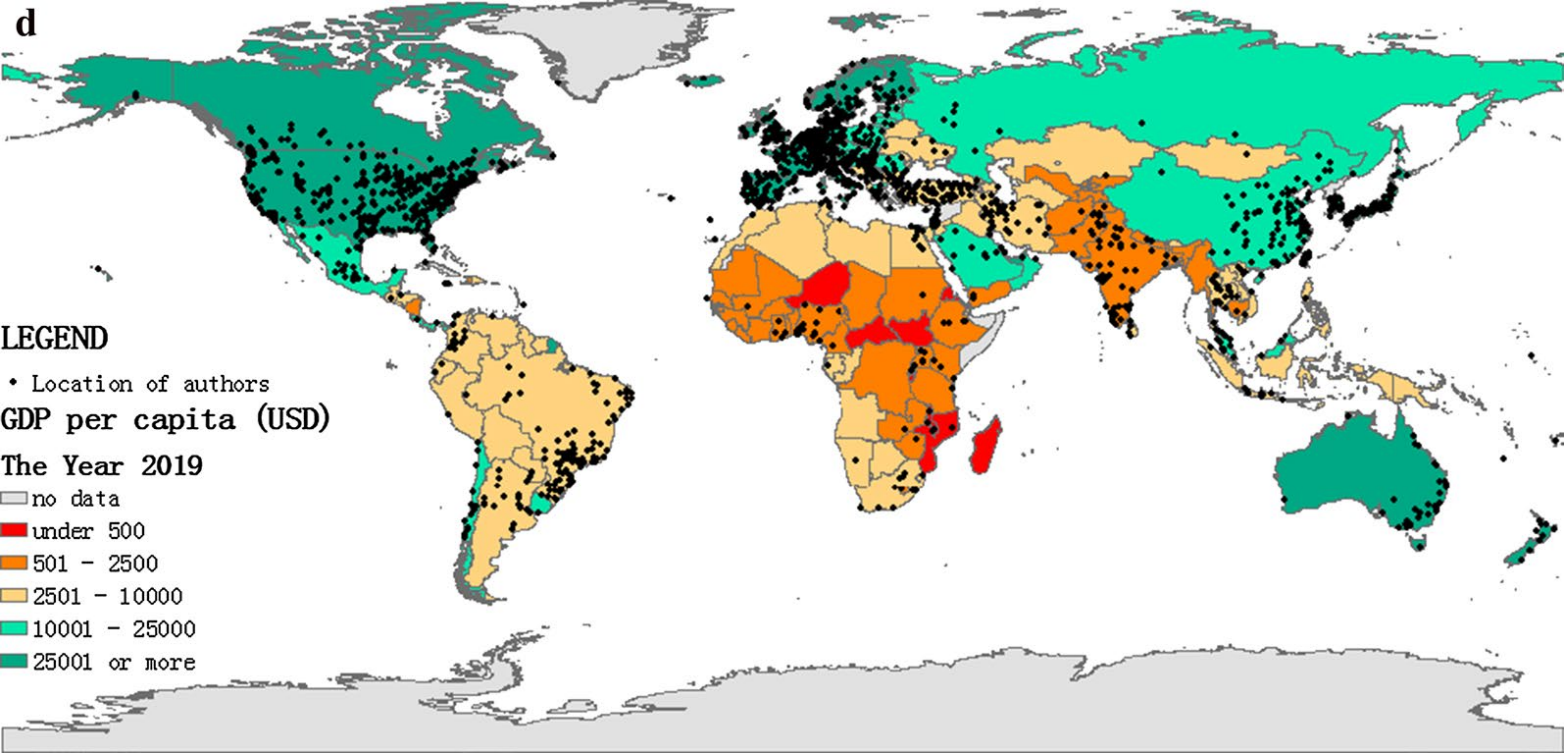
**a** Geographical distribution of authors (2000–2004). **b** Geographical distribution of authors (2005–2009). **c** Geographical distribution of authors (2010–2014). **d** Geographical distribution of authors (2015–2019)

In the collected data, 38 articles lacked the authors' addresses. Hence the geographic analysis included 25,081 items. We divided every five years as a period, and the background corresponded to each country's gross domestic product (GDP) per capita^1^ in the last year of each period. Furthermore, every dot represented a research institution to which the author belongs, as shown in Fig. 3a–d. In the first period (2000–2004), the main HRQoL research areas were distributed across North America and Europe. In the second period (2005–2009), besides North America and Europe, HRQoL research boomed in eastern Asia, southern Brazil, and Turkey. In the next period (2010–2014), China and India became newly-active areas in HRQoL research, and there was a small cluster of dots in eastern Australia, Western Asia, and Africa. In the last period (2015–2019), South America and Africa became more active than in previous periods, HRQoL ushered in a vigorous development worldwide in this period. However, clusters in Africa were more scattered. There was a regional disparity in the research on HRQoL.

At the country/territory level, there were 145 countries/territories participating in HRQoL research. The top 20 countries/territories with articles were listed in Table 2. The USA was the leading country in HRQoL research, published 30.37% of all articles, followed by UK (12.4%), Germany (9.22%), and Canada (8.04%). Also, international cooperation between different countries varied significantly. The USA, Germany, China, Netherlands, Sweden, Spain, Japan, Brazil, Norway, South Korea, Finland and Turkey preferred to give priority to independent research, the number of internationally-collaborative articles (CA) were all less than their number of single country articles (SA); UK, Canada, Australia, Italy, France, Switzerland, Denmark and Belgium seemed more inclined to cooperation.

**Table 2:** Top 20 most productive countries in HRQoL research

Country	TA (%)	SA (%)	CA (%)	SA/CA
USA	7628(30.37)	4945(64.83)	2683(35.17)	1.84
UK	3114(12.4)	1257(40.37)	1857(59.63)	0.68
Germany	2315(9.22)	1258(54.34)	1057(45.66)	1.19
Canada	2019(8.04)	893(44.23)	1126(55.77)	0.79
Netherlands	1911(7.61)	1133(59.29)	778(40.71)	1.46
Sweden	1541(6.13)	864(56.07)	677(43.93)	1.28
Spain	1537(6.12)	911(59.27)	626(40.73)	1.46
Australia	1380(5.49)	636(46.09)	744(53.91)	0.85
China	1242(4.94)	812(65.38)	430(34.62)	1.89
Italy	1148(4.57)	568(49.48)	580(50.52)	0.98
France	1006(4)	390(38.77)	616(61.23)	0.63
Japan	858(3.42)	643(74.94)	215(25.06)	2.99
Brazil	845(3.36)	556(65.8)	289(34.2)	1.92
Norway	701(2.79)	398(56.78)	303(43.94)	1.31
Switzerland	590(2.35)	131(22.2)	459(77.8)	0.29
South Korea	582(2.32)	447(76.8)	135(23.2)	3.31
Denmark	572(2.28)	230(40.21)	342(59.79)	0.67
Finland	559(2.23)	408(72.99)	151(27.01)	2.70
Belgium	479(1.91)	79(16.49)	400(83.51)	0.20
Turkey	470(1.87)	391(83.19)	79(16.81)	4.95

TA (%): the number of articles published by each country (percentage of all articles); SA (%): the number of single-country articles (percentage of TA); CA (%): the number of internationally-collaborative articles (percentage of TA)

**Fig. 4:**
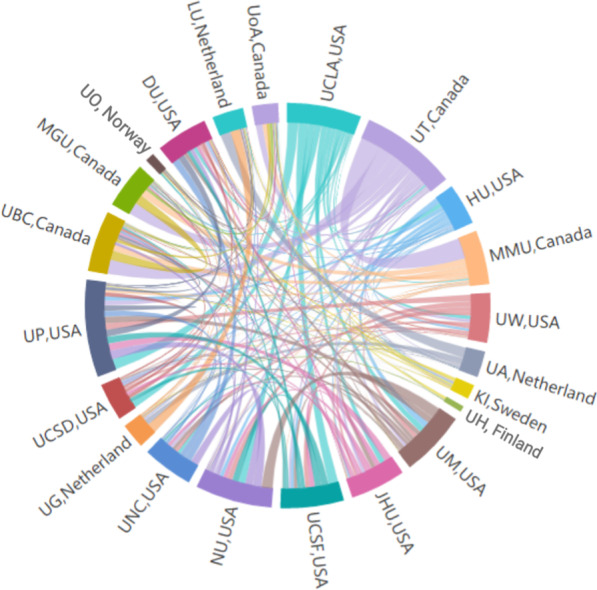
Chord diagram of the top 20 h-index institutions

At the institution level, we calculated the *h*-index in HRQoL research of each institution. The top 20 influential institutions were listed in Table 3 according to the rank of *h*-index, among the top 20 institutions, mostly in the USA(12), then in Canada(5). More important, The CA of these 20 institutions exceeded their SA. The CA of the University of Oslo, Norway, and Harvard University, USA reached more than 28 times of their SA, and the CA of the institution with the smallest gap also reached 4.9 times of the SA (University of North Carolina, USA). These results indicated that cooperative study between institutions was the main pattern in influential HRQoL research. Based on the visualization results of inter-institution collaboration (see as Fig. 4 in Appendix 2), we found that cooperation strength between domestic institutions was much greater than that of international institutions.

**Table 3:** Top 20 highest *h*-index institutions in HRQoL research

Institution	*h* (R)	TA (%)	SA (%)	CA (%)
University of California, Los Angeles *(UCLA)*,USA	63(1)	436(1.74)	39(8.94)	397(91.06)
University of Toronto*(UT)*, Canada	59(2)	543(2.16)	33(6.08)	510(93.92)
Harvard University*(HU)*, USA	57(3)	298(1.19)	10(3.36)	288(96.64)
McMaster University*(MMU)*, Canada	56(4)	312(1.24)	18(5.77)	294(94.23)
University of Washington*(UW)*, USA	51(5)	308(1.23)	23(7.47)	285(92.53)
University of Amsterdam*(UA)*, Netherlands	50(6)	294(1.17)	43(14.63)	251(85.37)
Karolinska Institute*(KI)*, Sweden	49(7)	425(1.69)	29(6.82)	396(93.18)
University of Helsinki*(UH)*, Finland	48(8)	325(1.29)	29(8.92)	296(91.08)
University of Michigan*(UM)*, USA	47(9)	339(1.35)	36(10.62)	303(89.38)
Johns Hopkins University*(JHU)*, USA	47(9)	266(1.06)	12(4.51)	254(95.49)
University of California, San Francisco *(UCSF)*,USA	46(11)	330(1.31)	26(7.88)	304(92.12)
Northwestern University*(NU)*, USA	45(12)	401(1.6)	24(5.99)	377(94.01)
University of North Carolina*(UNC)*, USA	45(12)	268(1.07)	45(16.79)	223(83.21)
University of Groningen*(UG)*, Netherlands	44(14)	312(1.24)	35(11.22)	277(88.78)
University of California, San Diego *(UCSD)*,USA	44(14)	199(0.79)	18(9.05)	181(90.95)
University of Pittsburgh*(UP)*, USA	43(16)	266(1.06)	28(10.53)	238(89.47)
University of British Columbia*(UBC)*, Canada	43(16)	258(1.03)	15(5.81)	243(94.19)
McGill University*(MGU)*, Canada	42(18)	240(0.96)	12(5)	228(95)
University of Oslo*(UO)*, Norway	41(19)	278(1.11)	9(3.24)	269(96.76)
Duke University*(DU)*, USA	40(20)	268(1.07)	16(5.97)	252(94.03)
Leiden University*(LU)*, Netherlands	40(20)	256(1.02)	33(12.89)	223(87.11)
University of Alberta*(UoA)*, Canada	40(20)	247(0.98)	28(11.34)	219(88.66)
University of Pennsylvania*(UP)*, USA	40(20)	233(0.93)	24(10.3)	209(89.7)

H Index(R): h index (rank); TA (%): the number of articles published by the institution (percentage of all articles); SA (%): the number of single-institution articles (percentage of TA); CA (%):inter-institutional collaborative articles (percentage of TA)

### Keyword analysis

According to the statistics of keywords, 25,191 keywords were used between 2000 and 2019, among which 19,155 keywords appeared only once or twice. After the keywords clustering work, we found that HRQoL research has six concentrated categories:

1. *HRQoL*, it was closely linked with terms like: "questionnaire", "SF-36", "EQ-5D", "patient-reported outcome", "children", "health status", "validity", "psychometrics", "clinical trial", "reliability", "patient satisfaction", "pedsql", "Parkinson's disease", "rheumatoid arthritis", "breast cancer", etc.
2. *Depression*, this category included terms such as "anxiety", "rehabilitation", "exercise", "elderly", "mental health", "social support", "cognition", "Parkinson’s disease", "stroke", "physical activity", "hemodialysis", "HIV ", "Epilepsy ", etc.
3. *Obesity*, with terms like "adolescent", "bariatric surgery", "weight loss", "children", "morbid obesity", "stress", "BMI", "physical activity", "gastric banding", "exercise", etc.
4. *Disability*, including terms such as "multiple sclerosis", "functional disability", "Oswestry Disability Index", "comorbidity", "pain", "migraine", "rheumatoid arthritis", "survival", "complication", etc.
5. *Oncology*, with "cancer", "breast cancer", "radiotherapy", "chemotherapy", "survivorship", "psychological distress" etc.
6. *Fatigue*, had "chronic fatigue syndrome", "cognition", "burden of illness", "anxiety", "sleep", etc. In addition, fatigue was a quickly rising term from 2000 to 2019.

Table 4 listed the top twenty quickly rising keywords sorted by their SGR 4. We chose SGR 4 because the terms selected according to the sudden increase in popularity were particularly appropriate to characterize a current research front [20]. All these twenty keywords besides "women" maintained a growing interest in HRQoL research from 2000 to 2019, "women" experienced a decline in period 3. What's more, these keywords had different growth rates in different periods. The growth in research interest for "dementia" had continued to accelerate over the past 20 years; the growth rate in research interest of "wellbeing", "surgery", "health", "HIV", "chronic disease", "aging", "women" and "stress" had slowed down in the period 3 (2010–2014), but had risen rapidly in period 4 (2015–2019). Therefore, we believed that the heat of these nine keywords might continue to increase in future research.

**Table 4:** Top 20 quickly rising keywords in period 4

Keyword	ASGR (R)	SGR 2	SGR 3	SGR 4
Older adults	2.92(6)	6.33	0.41	2.03
Dementia	0.99(41)	0.57	0.91	1.48
Cognition	1.54(14)	1.20	2.00	1.42
Patient-reported outcomes	3.26(4)	4.00	4.40	1.37
Survivorship	2.16(6)	2.50	2.86	1.11
Well-being	1.44(13)	2.80	0.47	1.04
Surgery	1.29(16)	2.25	0.69	0.93
Health	0.83(47)	1.44	0.15	0.91
Mental health	1.38(14)	1.50	1.77	0.88
HIV	1.06(30)	2.15	0.22	0.82
Pediatric	1.26(18)	1.60	1.38	0.81
Oral health-related quality of Life/OHRQoL	2.37(4)	5.46	0.89	0.76
Chronic disease	1.1(26)	2.43	0.13	0.74
Aging	0.71(51)	0.50	0.89	0.74
Women	0.79(42)	1.75	− 0.09	0.70
Stress	0.99(29)	1.67	0.63	0.69
Pain	1.14(22)	1.38	1.37	0.68
Fatigue	1.07(25)	1.71	0.82	0.66
Physical activity	1.55(9)	1.36	2.64	0.65
Sleep	1.13(21)	2.00	0.76	0.62

ASGR *(R): Average of SGR 2, SGR 3 and SGR 4 (rank); SGR 2, SGR 3 and SGR 4: the Sequential Growth Rate of period 2, period 3, and period 4

## Discussion

This study conducted a bibliometric analysis on HRQoL to provide some evidence on publications growth trends, geographic distribution, research trends, and other related bibliometric indicators. We will discuss the following aspects.

### Developmental tendency

The scientific production on HRQoL topics showed increasing development and institutional affluence. But it was worth noting that the TC and CPA were both in a decreasing trend, which may suggest that the research interest had gradually spread across multiple fields, consistent with the journal analysis. Another finding was that most of the most-cited articles were about HRQoL measurement instruments. (see as Table 5 in Appendix 1) Specific instruments for children [13, 21], for diseases like depression [22] and chronic obstructive pulmonary disease (COPD) [23], as well as generic instruments for the whole population [10, 24], the HRQoL measurement instruments achieved significant development between 2000 and 2011.

### Regional disparity

The geographical analysis results showed that HRQoL research in the past two decades was mainly distributed in North America and Europe. Research in some other regions like Asia, South America, and Africa was less, and the time of germination of research interest was later. Countries or regions with high GDP per capita had a higher scientific output on HRQoL research (Fig. 3a–d). Consistent with other studies, a country or region’s economic level have influence on its scientific output [25–27]. High-income countries in North America and Europe had more robust academic research capacity in HRQoL than low-income countries because they had numerous research institutions, well-established data systems, more research funds, and plenty of epidemiological research [28, 29]. What’s more, the policy and management of scientific research systems needs revision to harmonize with the national economic capacity, especially Russia. The research interest of HRQoL was affected by the internal health care system of the country or region. Health care systems in high-income countries such as the National Health Services (NHS) in the UK, the Managed Care in America, and the Regional Health Systems (RHS) in Singapore, these systems provided standardized medical procedures, comprehensive rehabilitation, and nursing services [30–32]. The demand for high-quality health care raised plenty of scientific questions about HRQoL. China and India launched national health care reforms in 2009 and 2010, setting a series of health policies trying to integrate health care services to improve the quality of medical care and the health outcome [33, 34]. The goals of health care reforms made HRQoL research topics more attractive, and research could receive more support, including data and funding. Besides, the model of collaboration between research institutions also had an impact on the regional scientific productivity [35, 36]. Existing academic collaboration models made it difficult for regions with low scientific productivity to obtain more scientific resources on HRQoL research. HRQoL issues in these areas may remain undiscovered and unstudied for a long time, which calls for future attention.

### Hotspots and future trend

A large number of author keywords were only used once or twice from 2000 to 2019, which indicated a lack of continuity in HRQoL research and a wide disparity in research focuses [37].In the future, introducing a set of general keywords should be encouraged to lead to a more uniform use of terms and definitions in the literature on HRQoL [38, 39].

From the keyword clusters, we can explore research hotspots mainly in three parts as follows:

### HRQoL instruments

HRQoL instruments can be separated into two main genres: generic measures intended to be appropriate for groups differing in disease, severity, and co-morbidity; specific measures designed to apply to particular patient groups or populations [4]. The most commonly used generic HRQoL instruments were SF-36, EQ-5D, SF-12; SF-6D; World Health Organization on Quality of Life Brief Scale (WHOQOL-BREF). WHOQOL-BREF was the shorter version of WHOQOL-100, [40] and SF-12 [41] and SF-6D [42]were derived from SF-36. These instruments were used in both general population and patient samples to estimate the relative burden of different diseases and conditions, and differentiate the health benefits produced by a wide range of different treatments. What's more, they were translated into different language versions and compared with other generic and disease-specific instruments [8, 10]. Besides, we identified the future need for developing brief, convenient, accurate, and cross-culturally applicable HRQoL instruments, especially for large-scale studies.

However, there was increasing demand for high-quality, specially designed questionnaires based on patient-reported outcomes (PROs) in clinical practice. The most commonly used specific HRQoL instruments were Pediatric Quality of Life Inventory (PedsQL), the European Organization for Research and Treatment of Cancer Quality of Life Core Questionnaire (EORTC QLQ-C30), the St. George's Respiratory Questionnaire (SGRQ), the Patient-Reported Outcomes Measurement Information System (PROMIS), The Oral Health Impact Profile (OHIP), and MOS-HIV. We noticed that there were still many diseases that have not developed their specific HRQoL instruments [43]. Small but significant changes in disease progression may be missed, which requires further study.

### Disease/conditions

HRQoL varied greatly between disease groups [44]. The most studied conditions and diseases among the past twenty years were depression, obesity, disability, cancer, fatigue, pain, multiple sclerosis, oral infections, stroke, asthma, COPD, Parkinson's disease, HIV, rheumatoid arthritis, and heart failure. HRQoL data provided scientific evidence for clinical decision-making and preventive health care. It helped find the patient-centered solutions for an evidence-based selection of optimal treatments, psychosocial interventions, patient-physician communications, and resource allocation to promote health and wellbeing [45–48].

As advances clinical treatment were realized, the number of survivors of certain diseases, such as cancer and AIDS, may increase over time, individuals may face the challenge of adding health to life years [22, 49, 50]. The increasing expectations of good HRQoL had led to increased concern about more health issues. Previous studies found oral disorders had a much broader impact on daily living than previously appreciated, especially for the elderly [51, 52]. The global prevalence of obesity had caused widespread concern. It was associated with higher fatigue rates, perceived stress, sleep disorders [53], and some other chronic diseases, which caused the decline in HRQoL directly and indirectly [54]. The Ageing of the world's population increased the prevalence of individual health conditions associated with older people [55, 56]. For instance, the increasing number of people with dementia posed many challenges to the health care system [56]. Future research needs to identify the comprehensive risk factors for HRQoL scores in these conditions. What's more, the clinical significance of changes in HRQoL scores may require attention, and it is suggested that there is a difference in the meaning or value patients place upon a change in HRQoL [57].

### Population characteristics

The HRQoL of the elderly, women, and children had received continuous attention over the past 20 years. The main factors affecting the HRQoL of these populations were demographic, sociological, lifestyle factors, as well as health status. Prior studies reported age and gender differences in subjective health and HRQoL in childhood, adolescence, and adulthood [58–60]. Factors such as education level [61], socioeconomic status [62–64], and social support [61, 65] also had an impact on HRQoL. Children's HRQoL was also affected by parental mental health, parent–child relationships [59, 63], and migration background [62]. Women experienced a higher prevalence of physical and mental health impairment than men, and they were twice as likely as men to experience depressive disorders [64]. Being married and having low perceived stress levels had a considerable positive impact on the HRQoL in women [61]. The leading health status associated with HRQoL of the elderly were: cardiovascular diseases, number of co-morbidities/diseases, functional incapacity, depressive symptoms, and cognitive function [60, 66]. What's more, living alone [67] and unhealthy lifestyles [60, 68]were associated with poor HRQoL in the elderly.

Previous studies had found that HRQoL improved after lifestyle modification [69]. A healthy diet, proper exercise, quitting cigarettes and alcohol effectively improved HRQoL [64, 69, 70]. Future research should focus on longitudinal studies to identify predictors of HRQoL that are amenable to intervention and should evaluate whether changes in these predictors result in more favorable HRQoL.

There were some limitations in this study. Firstly, in the data screening stage, we did not review all articles' abstracts because the sample size is too large, resulting in the omission of a small number of articles closely related to the research topic. Secondly, many institutions in the author's address used abbreviations, so that we lost a small part of the information when using Python to obtain the author's geographic data. Although we supplemented it with the manual query, there were still some omissions. Thirdly, before the keyword analysis, synonymous keywords were merged. Although the process was carried out in parallel by two authors and the results of each step were carefully compared, some merge errors and missed merges may still occur. The same problem may occur in the keyword clustering work. Finally, in the analysis of citations, there was no exclusion of self-citation, which might have some effect on the results.

## Conclusion

This study provided an overall perspective of global research trends and hotspots in HRQoL and potential future research insights, advised on practice and policy to promote the HRQoL research. HRQoL research had experienced significant increasing development during 2000–2019, especially the HRQoL measurement instruments. There were regional disparities in scientific output and collaboration, these regional differences may be associated with the economic level, health care systems, and academic collaboration models. The research hotspots and future research trends of HRQoL were mainly related to the HRQoL instruments, diseases/conditions, and particular populations.

## Data Availability

The datasets used and/or analysed during the current study are available from the corresponding author on reasonable request.

## Appendix 1

See Table 5.

**Table 5:** Top 10 most-cited articles (with citations ≥ 900)

Article title	TC(R)	PY	Publication name
PedsQL (TM) 4.0: Reliability and validity of the pediatric quality of life Inventory (TM) Version 4.0 generic core scales in healthy and patient populations	2313(1)	2001	Medical Care
Interpretation of changes in health-related quality of life—The remarkable universality of half a standard deviation	2212(2)	2003	Medical Care
Development and preliminary testing of the new five-level version of EQ-5D (EQ-5D-5L)	1628(3)	2011	Quality of Life Research
Quality of life and satisfaction with outcome among prostate-cancer survivors	1450(4)	2008	New England Journal of Medicine
The COSMIN study reached international consensus on taxonomy, terminology, and definitions of measurement properties for health-related patient-reported outcome	1190(5)	2010	Journal of Clinical Epidemiology
Development and first validation of the COPD Assessment Test	1123(6)	2009	European Respiratory Journal
The PedsQL (TM) 4.0 as a pediatric population health measure: Feasibility, reliability, and validity	1117(7)	2003	Ambulatory Pediatrics
The Patient-Reported Outcome Measurement Information System (PROMIS) progress of an NIH roadmap cooperative group during its first two years	1069(8)	2007	Medical Care
The PHQ-8 as a measure of current depression in the general population	1057(9)	2009	Journal of Affective Disorders
Health-related quality of life of severely obese children and adolescents	945(10)	2003	Journal of the American Medical Association

*TC(R)* total citations count (rank), *PY* published year

## Appendix 2

In Fig. 4, the sectors differ in color and area: a specific color corresponds to an institution, whereas its area is proportional to the volume of papers from that institution. Moreover, the thickness of links between sectors represents the strength of cooperation among the institutions.

## Abbreviations

HRQoL: Health-related quality of life
WOS: Web of science
TC: Total citations
CPA: Citations per article
QoL: Quality of Life
EQ-5D: EuroQol five dimensions questionnaire
SF-36: 36-Item short-form
IQOLA: International quality of life assessment
IF: Impact factor
SCIE: Science citation index expanded
SSCI: Social sciences citation index
SGR: Sequential growth rate
AAP: The average number of authors per article
ARP: The average number of references cited per article
APP: The average number of pages per article
GDP: Gross domestic product
CA: Internationally-collaborative articles
SA: Single country articles
UCLA: University of California, Los Angeles
COPD: Chronic obstructive pulmonary disease
OHRQoL: The oral health related quality of life
PROs: Patient-reported outcomes
NHS: National Health Services
RHS: Regional Health Systems
EORTC QLQ-C30: European organization for research and treatment of cancer quality of life core questionnaire
SGRQ: St. George's respiratory questionnaire
PROMIS: Patient-reported outcomes measurement information System
OHIP: Oral health impact profile
MOS-HIV: Medical outcomes study HIV
WHOQOL-BREF: World health organization on quality of life brief scale
PedsQL: Pediatric quality of life inventory

## Footnotes

1 The GDP per capita data come from “World Economic Outlook Database, October 2019”.
